# The Role of the *GSTF11* Gene in Resistance to Powdery Mildew Infection and Cold Stress

**DOI:** 10.3390/plants10122729

**Published:** 2021-12-11

**Authors:** Elena Mikhaylova, Emil Khusnutdinov, Michael Yu Shein, Valentin Yu Alekseev, Yuri Nikonorov, Bulat Kuluev

**Affiliations:** Institute of Biochemistry and Genetics UFRC RAS, Prospekt Oktyabrya 71, 450054 Ufa, Russia; emil.khusnutdinov.18@bk.ru (E.K.); mikeshenoda@yandex.ru (M.Y.S.); valentin-1994@yandex.ru (V.Y.A.); nikonorov@anrb.ru (Y.N.); kuluev@bk.ru (B.K.)

**Keywords:** *Brassica napus*, oilseed rape, rapeseed, powdery mildew, ITS, *Erysiphe crucifertaum*, GST, GSH, GSSG, glucosinolates, ITC, oxidative stress

## Abstract

Oilseed rape (*Brassica napus*) is an economically important crop. In a temperate climate, powdery mildew *Erysiphe crucifertaum* can drastically reduce its yield. Nevertheless, cultivars resistant to this fungal disease have not yet been selected. Glutathione S-transferase GSTF11 is involved in glucosinolate (GSL) biosynthesis and response to stress, including fungal deceases. However, the impact of exogenous *GSTF11* gene expression on resistance to powdery mildew has not yet been confirmed and requires further investigation. Transgenic *B. napus* was generated for this purpose. It demonstrated increased GST activity and a higher GSH:GSSG ratio under normal conditions. Powdery mildew *Erysiphe crucifertaum* caused 50% mortality in wild type (WT) plants. In most of transgenic plants, mycelium growth was inhibited. The infection contributed to higher *GSTF11* expression and increased levels of glutathione (GSH) and oxidized glutathione (GSSG) in both transgenic and WT plants. In contrast, *GSTF11* mRNA content, GST activity and GSSG level were lower only in WT plants. In transgenic plants, increased resistance to powdery mildew correlated with a lower GSH:GSSG ratio, indicating a higher content of neutralized toxic molecules. *GSTF11* expression was also affected by cold stress, but not drought. At −1 °C, the expression level increased only in transgenic plants. Therefore, *GSTF11* appears to be nonspecific and is able to protect plants under several types of stress. This gene could be used as a target in the production of stress tolerant cultivars.

## 1. Introduction

*Brassica napus* L. (oilseed rape, rapeseed) is a source of vegetable oil and protein, animal feed and biodiesel. It is one of the most important crops in the temperate zone because of its remarkable cold tolerance. However, oilseed rape is highly susceptible to fungal diseases, including powdery mildew *Erysiphe crucifertaum* [1].

Powdery mildew is an obligate biotrophic pathogen, most frequently encountered at the end of the growing season [1]. The cleistothecia and mycelium of these fungi can overwinter in leaf debris and cause primary infection [2,3,4]. Ascospores produce hyphae that penetrate plant tissues. Conidia develop from hyphae and cause secondary infection. Symptoms develop faster in older plants [1]. Powdery mildew uses effector proteins to suppress plant defense responses and induce nitrate transport activity to acquire nutrients from the host. Recognition of such effectors triggers plant immune response, including cellular suicide [5]. Powdery mildew can reduce the yield and quality of rapeseed seeds, causing chlorosis, necrosis and dehydration. Resistance to this pathogen in *B. napus* is very rare, and was achieved only by hybridization with distant relatives [6]. Resistant commercial cultivars have not yet been selected. The impossibility of growing *E. crucifertaum* in a nutrient medium separately from the plant complicates the development of resistant cultivars.

The physiological responses of plants to powdery mildew and the mechanisms of resistance to this infection are diverse. For example, in tomatoes, six resistance genes mediate different types of responses to *Oidium neolycopersici* [7]. The “rapid” mechanism of resistance includes the immediate death of cells invaded by fungi. During the “slow” mechanism, cells invaded by primary haustoria remain alive, and cell death occurs only after invasion of secondary haustoria. Infection often causes an accumulation of reactive oxygen species (ROS) in resistant plants [7].

Glutathione S-transferases (GSTs), capable of binding a large number of endogenous and exogenous compounds, catalyze the detoxification of toxic molecules by reduced GSH. During this reaction, GSSG is formed [8]. The induction of GSH and GST levels upon exposure to stress provides better protection of plant cells. Although GSTs are usually associated with tolerance to heavy metals [9], there is evidence that several GST genes play a role in resistance to other stress factors such as herbicides [10], drought [11], extreme temperatures and salinity [12,13,14], viral and fungal deceases [7,15,16,17,18]. Several GST genes are specifically upregulated by fungal infections, including powdery mildew. They are associated with a “slow” defense mechanism and the ability to prevent excessive cell death [18]. In wheat, GST proteins of the class phi inhibited the toxicity of ROS and protected cells from oxidative stress [19]. In resistant wheat, the expression of the *GSTF5* gene was induced [20], and the expression of the *GSTA1* gene encoding the GST29 protein increased 20-fold [15,21]. Powdery mildew elevated the expression of the *GST1* gene [22] and the *GSTF6* gene [10] in *A. thaliana*. Knockout of a GST gene in resistant tomato produced a susceptible phenotype and allowed fungal growth and sporulation. The cells of resistant plants invaded by fungi rapidly accumulated H_2_O_2_ and died; however, the ratio of dead cells in susceptible plants was lower [7].

It is worth noting that some experiments have indicated lower GST activity in resistant plants. For example, transgenic barley, producing an Mtk antifungal peptide from fruit fly, had an elevated expression level of GST-6 gene. However, after infection with powdery mildew, GST-6 transcript level decreased [23]. In barley, a greater increase in GST activity upon infection was observed in susceptible cultivars [18].

Another mechanism of resistance to powdery mildew is associated with the formation of GSLs in *Brassicaceae* (including *B. napus* and the model plant *A. thaliana*). It has been suggested that the conjugation of GSH and GSTs is required for the biosynthesis of GSLs, as well as for the deactivation of isothiocyanates (ITCs), i.e., toxic GSL derivatives [24]. Derivatives of GSL, synthesized upon tissue damage, provide protection against biotic stress, especially against fungi. Powdery mildew induces the expression of cytochrome monooxygenase genes involved in the biosynthesis of aliphatic GSL and the accumulation of GSL biosynthetic enzymes in epidermal cells of *A. thaliana* [25,26,27].

Despite protection from fungi, ITCs act as herbicides, causing the loss of chlorophyll, oxidative stress, and a decrease in the GSH content in the plant itself [28,29,30,31,32]. *GST* genes are upregulated in response to isothiocyanates, providing rapid detoxification to recover from reduced glutathione (GSH) levels [33].

Powdery mildew resistance is also regulated by the *Mlo* genes associated with the papilla response and rapid cell death. The loss-of-function mutation prevents fungal penetration attempts and promotes suicide response in attacked barley cells. The increase in the frequency and diameter of the papillae contribute to the resistance to powdery mildew infection [29,34,35]. In *Arabidopsis thaliana*, this kind of resistance appears to be polygenic [22,29,36].

Thus, we suggest that GSTs may be the key molecules involved in at least two protection mechanisms during plant–fungus interactions. There are 48 GST-like genes in the genome of *A. thaliana*. Among them, *GSTF11* stands out for its high homology with the *GST* genes of the genus *Brassica*, which is rich in GSLs. It has been demonstrated that recombinant *AtGSTF11* expressed in *E. coli* was most active with benzyl isothiocyanate (BITC) as a substrate [10,24]. BITC is a pathogen-triggered derivative of GSL that is toxic to arthropods, nematodes and fungi [37]. However, the *GSTF11* gene is not yet associated with the production of precise GSL, and sometimes it does not respond to infection or ITC treatment [24,31,38,39]. It is assumed that in *B. napus GSTF11* acts as one of the core GSL biosynthesis genes. However, upon stem rot infection caused by *Sclerotinia sclerotiorum*, this gene was downregulated [40].

*GSTF11* has been also associated with response to other types of stress, but the data are contradictory. Transgenic hairy roots of tobacco demonstrated increased tolerance to salinity, heat stress and heavy metal pollution due to At*GSTF11* expression [41]. This gene also enhances anthocyanin pigmentation in ornamental crop *Euphorbia pulcherrima* [42]. The *GSTF11* gene of *Arabidopsis* is closely related to Transparent testa *(TT19)*, which uses anthocyanins as substrates and participates in the proanthocyanidin pathway [43]. Therefore, the functions of the *GSTF11* gene require verification.

Nevertheless, GSTs appear to have a unique function in *Brassicaceae* due to the occurrence of GSLs in these plants. Exogenous expression of the *AtGSTF11* gene has never been researched in related plant species. The aim of this study was to provide insights into the role of GSTF11 in response to various types of stress. To investigate the prospects for increasing plant resistance to stress via overexpression of the *AtGST11* gene, a transgenic *B. napus* was generated, and its response to powdery mildew, cold and drought stress was evaluated.

## 2. Results

### 2.1. Characteristics of Transgenic Plants

Under normal conditions, the level of expression of the *GSTF11* gene in *A. thaliana* was 12% of the reference genes *Actin7* and *UBC9*; however, in WT *B. napus*, the expression level of the endogenous *GSTF11* was 2% of the same reference genes. In transgenic *B. napus*, the expression level of *GSTF11* increased only up to 4.3% under normal conditions. Basic morphological parameters of transgenic plants, except for the stem length, did not change significantly. The normal range of stem length for the cultivar “Ratnik” is 84–119 cm. The stem length of transgenic plants was only 50 ± 8.56 cm. The stem length in WT plants was 82 ± 8 cm.

A higher *GSTF11* mRNA content in transgenic plants was accompanied by an increase in the activity of the GST enzyme by an average of 30%. The average GST activity in transgenic plants was 255 μg/min/mg protein in laboratory conditions and 454 μg/min/mg protein in the experimental plot.

### 2.2. Identification of Pathogen

**Figure 1:**
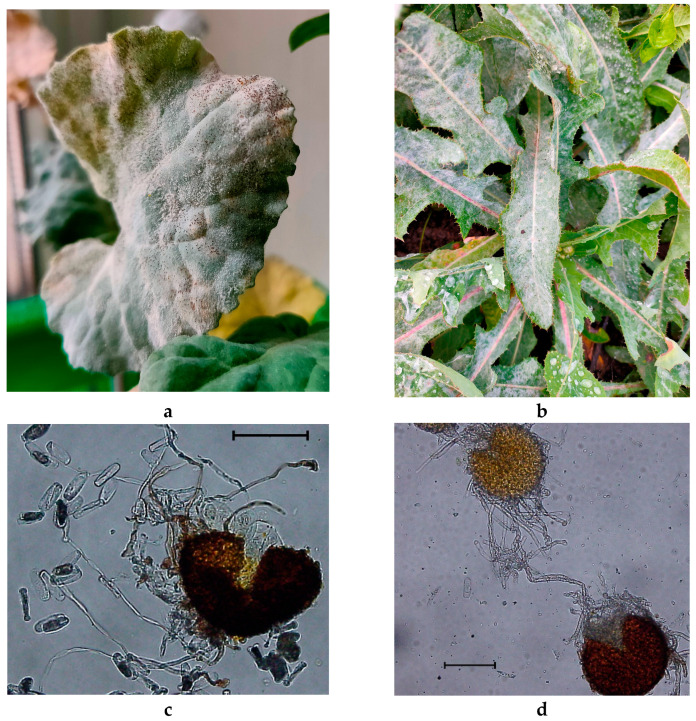
Powdery mildew in: *B. napus* (**a**,**c**) and *S. oleraceus* (**b**,**d**). The scale corresponds to 100 µm.

A microscopic analysis of the lavage from the leaves of *B. napus*, *Convolvulus arvensis* and *Sonchus oleraceus*, covered with white powdery growth, allowed to identify spores and chasmothecia characteristic of *Erysiphaceae* (see Figure 1). It should be noted that under natural conditions, chasmothecia were not detected in *C. arvensis* and *B. napus*. However, in the laboratory, these structures were produced by each specimen of powdery mildew.

**Figure 2:**
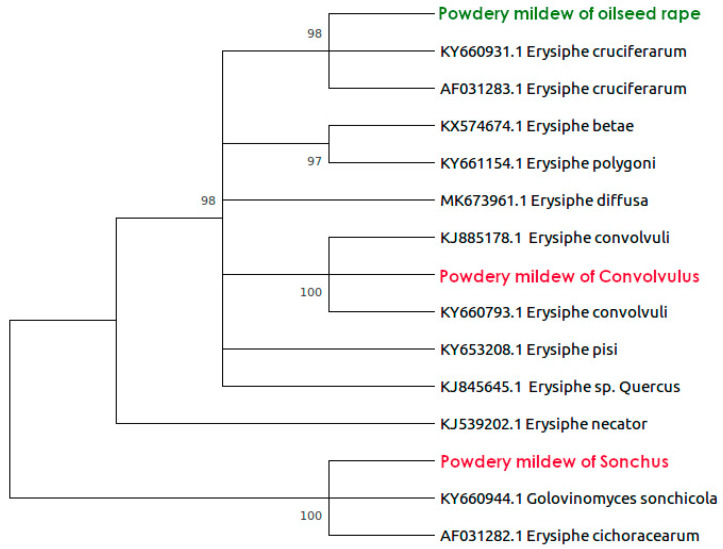
Phylogenetic maximum-likehood tree of *E. crucifertaum, E. cichoracearum* and *E. convolvuli* (highlighted in color). Bootstrap values >70% computed by 1000 replicates are given.

According to the sequencing results, the amplified genetic marker was 959 bp in length and contained the internal transcribed spacer regions ITS1 and ITS2. Powdery mildew collected from *B. napus* demonstrated the greatest similarity with European isolates of *Erysiphe crucifertaum* (AF031283, KY660931) from the United Kingdom [15]. The pathogen from *C. arvensis* belonged to the *Erysiphe convolvuli* clade, and pathogen from *S. oleraceus* belonged to *Erysiphe cichoracearum* clade with a high level of support (see Figure 2). Thus, isolates of powdery mildew from an agricultural crop *B. napus* and its weeds clearly belong to different species. The sequences are available in the NCBI database (MW267299-MW267301).

### 2.3. Reaction of Transgenic Plants to Powdery Mildew

Signs of infection became clearly visible on *B. napus* 30 days after treatment with powdery mildew collected from plants of the same species. White powdery growth appeared mainly on older leaves. Powdery mildew collected from *S. oleraceus* and *C. arvensis* did not infect *B. napus*. This result suggests that this pathogen is species-specific.

**Figure 3:**
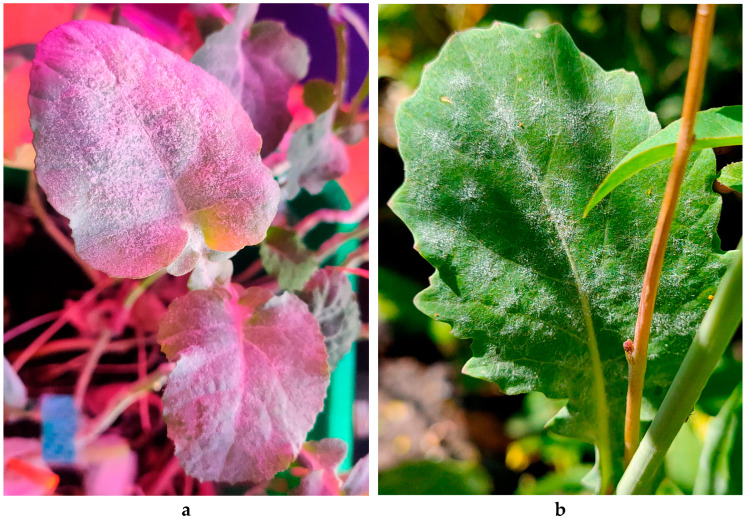

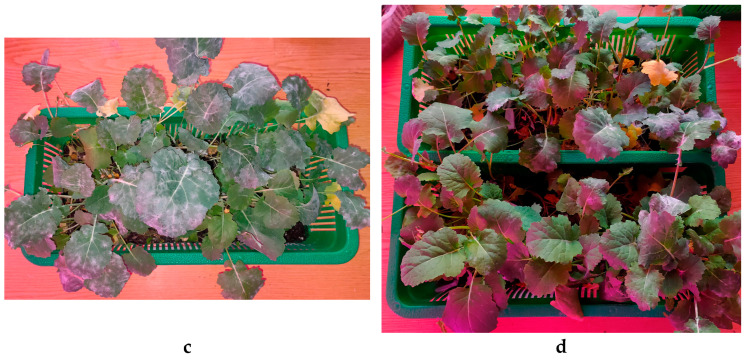
Degree of infection on WT *B. napus* (**a**,**c**) and transgenic plants (**b**,**d**).

The severity of infection was much higher in WT plants (60–70%) than in transgenic plants (30–40%) (see Figure 3).

Plants with less than 30% coverage with white powdery growth were organized into a “resistant” group. Plants with 60% and more coverage were organized into a “susceptible” group [44] to investigate the reasons of resistance. Transgenic plants from laboratory and experimental plot were analyzed separately. No plant was free of infection; however, 84% of WT plants and only 23% of transgenic plants were severely infected (Figure 3). Samples from all groups were subjected to real time PCR (RT-PCR) analysis, GST activity analysis, GSH and GSSG assays.

**Figure 4:**
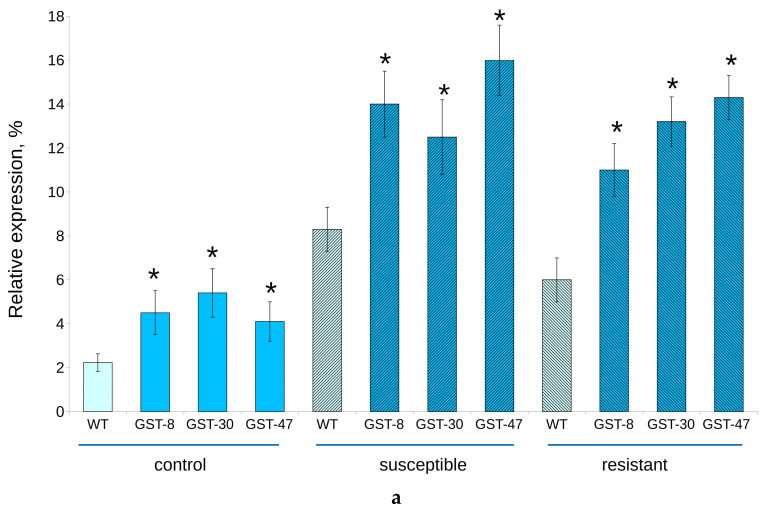

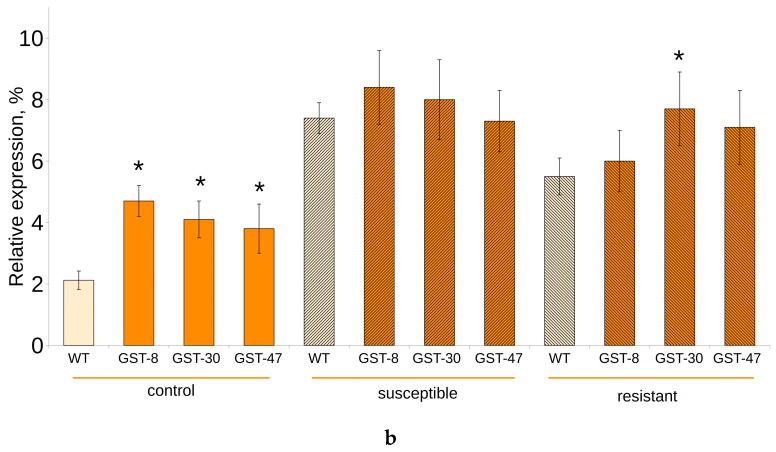
Relative expression level of *GSTF11* gene in transgenic (GST, lines 8, 30 and 47) and wild type (WT) *B. napus* exposed to powdery mildew infection. (**a**) Experimental plot; (**b**) Laboratory. Asterisk (*) indicates a significant difference between WT and transgenic plants from the same group.

The *GSTF11* expression level significantly increased after infection with powdery mildew, and was maximal in “susceptible” groups (Figure 4). The average content of *GSTF11* mRNA in transgenic plants reached 13.7% in the plot experiment and 8.2% in the laboratory experiment. Target gene expression was significantly higher in transgenic plants than in WT in the plot experiment; however, in the laboratory, there was no significant difference between transgenic and WT plants.

**Figure 5:**
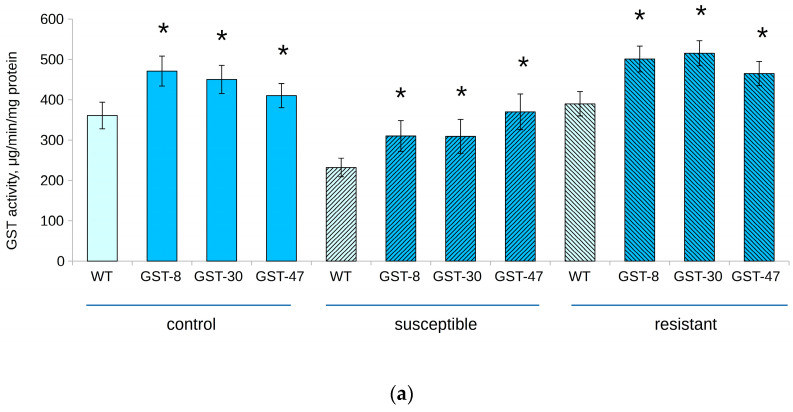

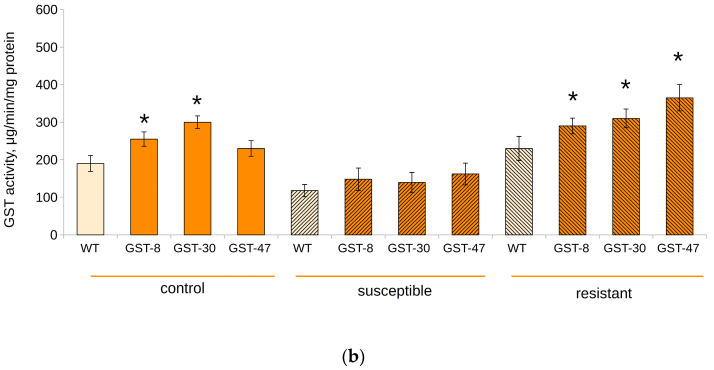
GST activity in transgenic (GST, lines 8, 30 and 47) and WT *B. napus*, exposed to powdery mildew infection. (**a**) Experimental plot; (**b**) Laboratory. Asterisk (*) indicates a significant difference between WT and transgenic plants from the same group.

Surprisingly, the lowest GST activity was detected in the “susceptible” groups, despite the higher content of *GSTF11* mRNA (Figure 5). In particular, the average value of this parameter in transgenic plants was 320 and 149 μg/min/mg protein in plot and laboratory experiments, respectively. The highest GST activity was detected in resistant transgenic plants (500 and 300 μg/min/mg protein). However, there was no significant difference between “resistant” and control groups. It is interesting to note that in the experimental plot, GST activity in transgenic and WT plants was approximately 50% higher than in the laboratory experiment.

The content of GSH increased dramatically in transgenic plants infected with powdery mildew. The highest values were observed in the “resistant” group. The average GSH content in transgenic plants increased from 25 to 750 nmol/g of fresh weight (FW) in the plot experiment and from 87 to 420 nmol/g FW in the laboratory experiment. The same trend was observed for the GSSG content. Its average value in transgenic plants increased from 0.3 to 14 nmol/g FW in the plot experiment and from 1.25 to 15 nmol/g FW in the laboratory experiment. The GSSG content in susceptible transgenic plants was half that observed in the “resistant” group (6.2 and 8 nmol/g FW in plot and laboratory experiments, respectively).

**Figure 6:**
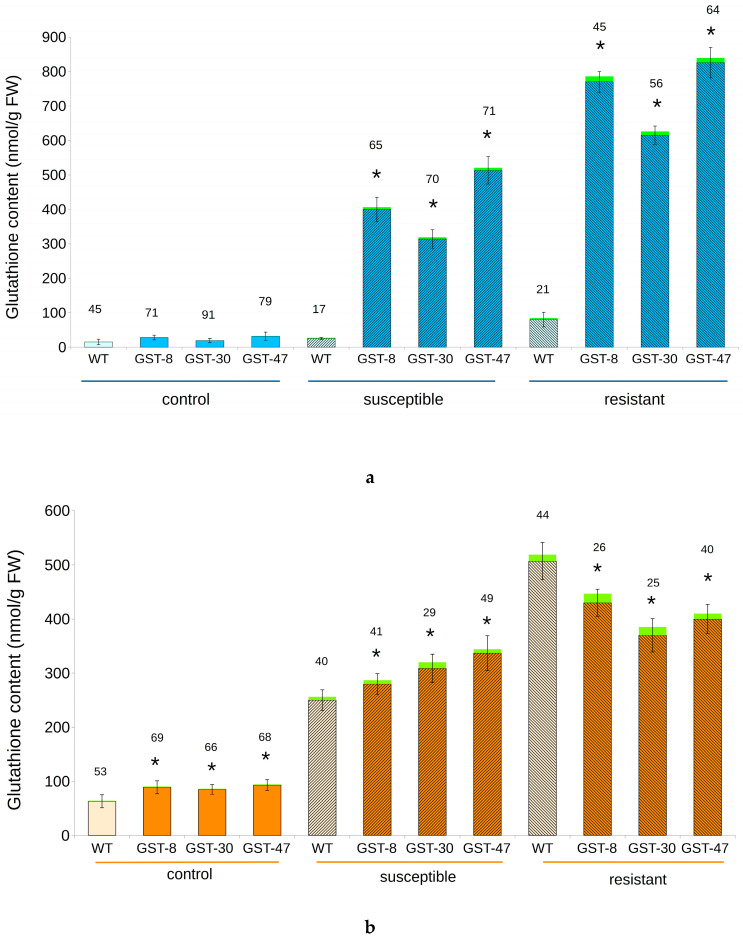
Content of total glutathione, including GSH (blue and orange) and GSSG (green) in transgenic (GST) and WT *B. napus* exposed to powdery mildew infection. The GSH:GSSG ratio is indicated above each table. (**a**) Experimental plot; (**b**) Laboratory. Visible error bars are calculated for GSH content. Asterisk (*) indicates a significant difference between WT and transgenic plants from the same group.

The GSH:GSSG ratio was higher in untreated transgenic plants, but after infection, this parameter decreased in all groups, especially in resistant plants. While in the experimental plot, nontransgenic plants demonstrated a weaker reaction, in the laboratory, the GSH content in WT and transgenic plants was comparable (Figure 6).

**Figure 7:**
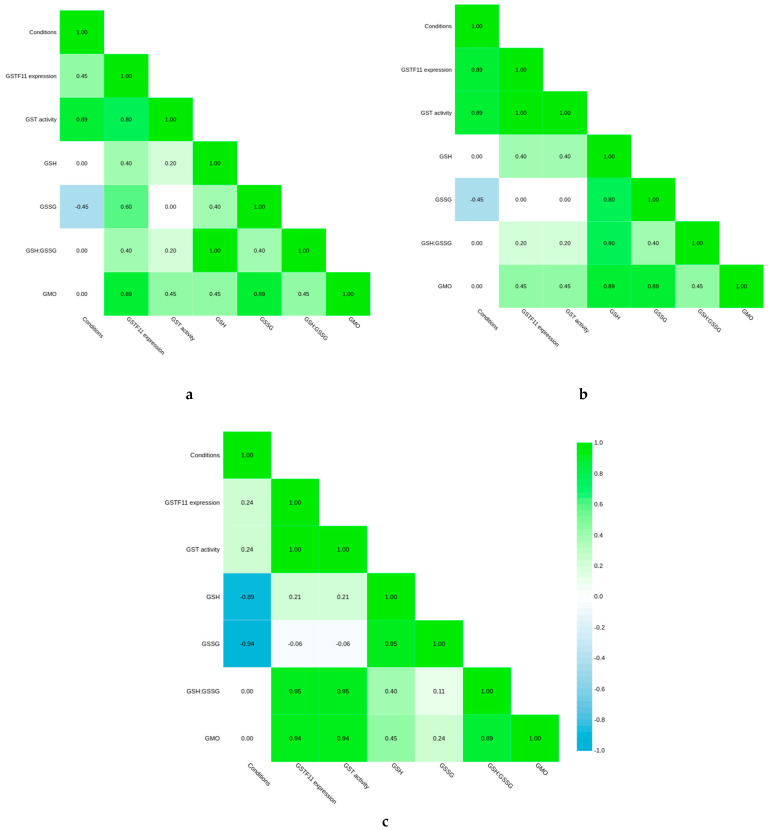
Correlation matrix representing the correlation between the transgene presence (GMO), *GSTF11* gene expression, experimental conditions and physiological parameters in (**a**) Resistant plants; (**b**) Susceptible plants; (**c**) Untreated plants.

In all groups, there was a strong correlation between *GSTF11* expression and GST activity. Untreated transgenic plants demonstrated increased GST activity, while there was no direct correlation between GST activity and the presence of an exogenous gene in infected plants. After infection with powdery mildew, transgenic plants accumulated more GSSG than WT. In the “susceptible” group, the presence of the transgene was associated with the GSH level and did not significantly correlate with *GSTF11* expression, which was highly dependent on cultivation conditions (Figure 7b).

In the “resistant” group, only the GST activity depended on the cultivation conditions, while untreated plants grown in the experimental plot were characterized by a lower GSH and GSSG content (Figure 7c).

Mortality among nontransgenic plants was 50%. Among transgenic plants, only 34% died after 100 days of cultivation.

### 2.4. Response of Transgenic Plants to Drought and Cold Stress

*GSTF11* is associated with a response to several types of stress [41,42,43]. To determine whether this gene is involved in general stress response or a specific response, transgenic plants were subjected to additional treatments.

**Figure 8:**
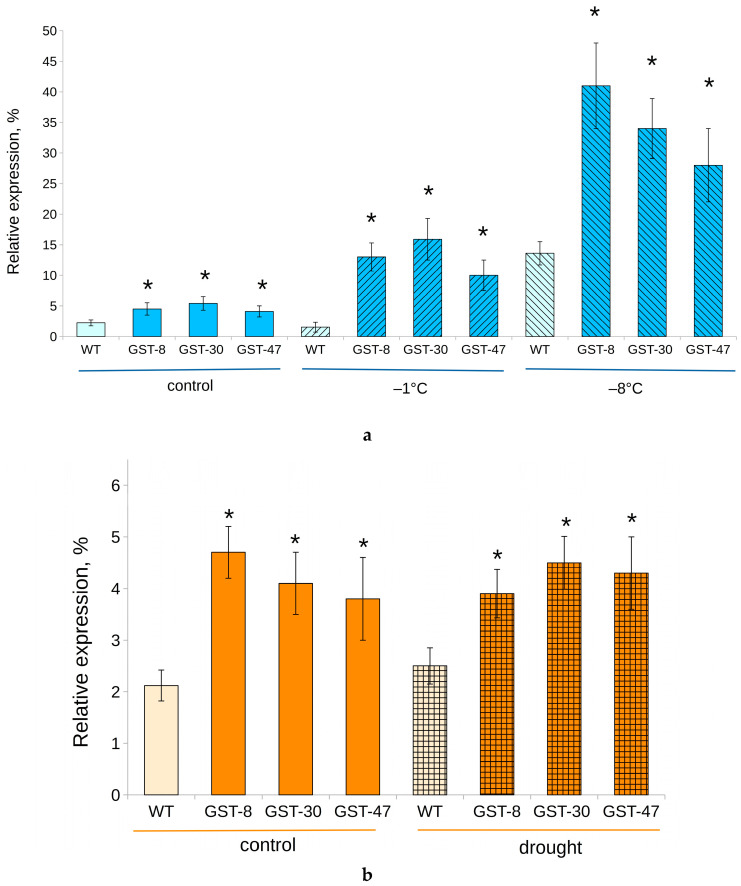
Relative level of expression of *GSTF11* gene in transgenic (GST, lines 8, 30 and 47) and WT *B. napus*. (**a**) Cold stress; (**b**) Drought stress. Asterisk (*) indicates a significant difference between WT and transgenic plants from the same group.

Transgenic plants subjected to drought stress were at the same stage of wilting as WT plants after 7 days of water depletion. After treatment, the content of *GSTF11* gene transcripts did not change significantly in either group (Figure 8b).

Cold stress contributed to a dramatic increase in the expression of *GSTF11* at −8°C, up to an average of 34.4% (Figure 8a). At −1°C, the *GSTF11* mRNA content was elevated in transgenic plants (to 12.5%), but decreased in nontransgenic plants.

It should be noted that expression of the reference gene *Actin7* was not affected by stress; in all samples, the reaction crossed the fluorescence threshold in cycles 25–26. Conversely, the expression of *UBC9* was significantly reduced at −8°C (30–32 cycles to cross the threshold). Heat and powdery mildew did not affect the expression of reference genes.

**Figure 9:**
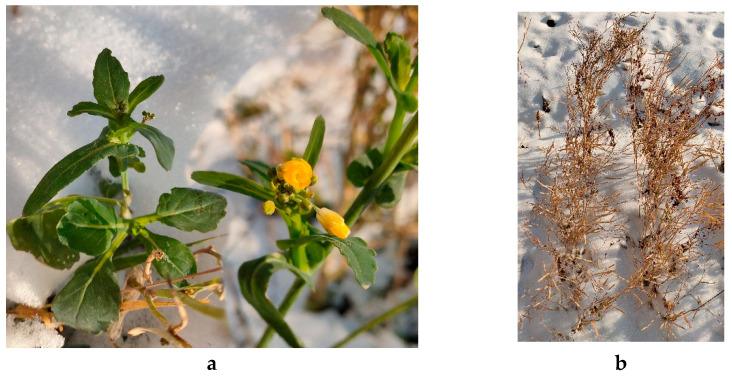
Transgenic (**a**) and WT plants (**b**) in experimental plot in November.

In the conditions of ground frost in November, some of the transgenic plants continued flowering and maintained greenery (Figure 9a); however, most of the WT plants withered (Figure 9b). Not a single plant withstood continual subzero temperature, and by the beginning of December, all plants were dead.

## 3. Discussion

In the present study, the involvement of the *GSTF11* gene in response to powdery mildew infection and cold stress was demonstrated in *B. napus* for the first time. It has been shown that in *B. napus*, this gene is not responsive to drought. Transgenic plants were characterized by increased resistance to *E. cruciferatum*, manifesting in smaller coverage with white powdery growth and lower death rate. Nevertheless, not a single plant was fully resistant, and the pathogen was able to complete the infection cycle. Mutations in *Mlo* genes [29,35] and cytochrome P450 monooxygenase gene CYP83A1, which is involved in aliphatic GSL biosynthesis [26,27], promoted stronger resistance. Nevertheless, *GSTF11* is still a promising target for the production of stress-resistant cultivars. In *Brassicaceae*, GSTs could increase the efficiency of these mutations.

In our experiments, higher *GSTF11* mRNA content was associated with resistance of transgenic plants to subzero temperatures, but not drought. Indeed, GSL production, GST activity and GSH content were induced in *Brassicaceae* during cold stress [45]. Conversely, drought downregulated GSL metabolism in *Brassica rapa* [46]. This is in agreement with our results, and explains the absence of drought resistance in the 35S:GSTF11 *B. napus* and rigidity of *GSTF11* gene expression (Figure 8b).

Based on research results and literature data, we suggest that GSTs provide a connection between two known mechanisms: a “slow” defense mechanism of binding toxic molecules by GSH [7,8,9,10,11,12,13,14,15,16,17,18,19,20,21,22], and a unique GSL-mediated protection mechanism of *Brassicaceae* [24,25,26,27]. GSTF11 may concurrently promote GSL biosynthesis and bind excessive GSL derivatives in the cell. When ITCs are synthesized upon powdery mildew infection, GSTs, including GSTF11, may protect plant cells and increase antifungal effect at the same time.

This possible mechanism requires further investigation; however, it may be difficult to prove experimentally, as information on the exact substrates for GSTs is contradictory. It have been shown that *GSTU5*, *GSTU13*, and *GSTF8* genes were upregulated by allyl ITC, and *GSTU19*, *GSTF6*, *GSTF7*, and *GSTZ1*—by phenethyl ITC. However, *GSTF11* expression was not affected by any of them [31]. In other studies GSTF11 and GSTU20 have been associated with aliphatic GSLs, GSTF9 and GSTF10—with indolic GSLs, and GSTF6—with camalexin biosynthesis [24,39,47]. Higher expression level of *GSTF11* gene have been associated with production of indolic GSLs and benzyl GSLs in *Nicotiana benthamiana* [38]. Exogenous *GSTF11* promoted glucoraphanin accumulation in transgenic N. benthamiana [39]. The results regarding the activity of GSTF11 with BITC as a substrate were obtained only in ex planta experiments [10,24]. These suggestions were not experimentally validated by the measurements of GSL derivatives concentration in plant tissues.

The fungicide activity of ITCs have been studied on different pathogens. Pure ITCs have been found to inhibit either conidia germination or mycelial growth, and sometimes both. Extracts from *Brassicaceae* plants inhibit only mycelial growth [30]. The degree of mycelial colony expansion on the leaves is usually measured and used as the only indicator of resistance to powdery mildew [1,44,48,49]. Fungicidal or fungistatic activity depends on ITC concentration [50,51]. The treatment of crops with *Brassicaceae* oil containing ITCs has been associated with the distortion of the powdery mildew conidia, although this did not affect their germination [48]. Therefore, an increased concentration of a specific ITC could account for inhibited mycelial growth in the 35S:GSTF11 *B. napus*. Most likely, the transgenic plants were not completely resistant, since ITCs could not suppress the conidial germination.

Since this ITC has not yet been identified, we used indirect methods to study the role of GSTF11. Resistance to stress is most often characterized by a higher ratio, and susceptibility by a lower ratio, of GSH:GSSG [52,53]. However, upon powdery mildew infection, transgenic *B. napus* produced more GSSG than WT, and therefore, had a lower GSH:GSSG ratio. This indicates that excessive GSTs bound to an unknown substrate that was present in the cell upon powdery mildew infection. This type of reaction was previously found in transgenic tobacco overexpressing GSTs with glutathione peroxidase activity [13,14], and was associated with increased resistance to stress. GSSG level was also elevated in untreated transgenic tobacco. However, in our experiments, the GSH:GSSG ratio was higher in untreated transgenic plants than in WT, despite increased GST activity. This could indicate that, without stress, the substrate of GSTF11 is absent in the cell or its content is low. It is known that glucosinolates are converted to ITCs only upon tissue damage, such as fungal penetration. Many experiments have reliably demonstrated the involvement of GSTF11 protein with ITCs, produced in response to biotic stress [24,28,33]. We propose that in the present study, ITC could also have served as a substrate for GSTF11. However, as long as the tissues remain intact, GSTF11 is likely to be involved in other processes. There is evidence of the binding of GSTF2, GSTF8, GSTF10 and GSTF11 with the key defense hormone, salicylic acid [18,54,55], but this mechanism remains unexplored. Since salicylic acid can improve the stem length of a crop, its interaction with excessive GSTF11 may explain the smaller stem lengths in transgenic plants in the present study. GSTF11 may also be involved in plant coloration [42,43].

In our experiments, the GSH level increased upon infection with powdery mildew in all groups. The most dramatic increase was observed in transgenic plants under experimental plot conditions. Moreover, in the “susceptible” group, the presence of the transgene was associated with increased GSH level. It has been demonstrated that ITCs can cause a depletion of the total glutathione pool [56]. However, *GST* genes can contribute to an increase not only in GST activity, but also in GSH content [52]. Hence, GSTF11 may also be involved in maintaining GSH level. It should be noted that several types of stress factors, such as cadmium, also contribute to a decrease in GSH level in susceptible *B. napus* [53].

Our results show that the GST activity in all groups correlated with *GSTF11* expression, indicating that 1-Chloro-2,4-dinitrobenzene (CDNB) is a suitable substrate for measuring GSTF11 activity. Nevertheless, the GST activity in severely infected plants was lower than in resistant plants, despite higher *GSTF11* gene expression. This parameter could be affected by a decrease in the content of other GSTs, since the activity of the entire pool of GST proteins was measured [37,57], as well as an increase in the protein content. The sample could contain various types of plant defense proteins, as well as proteins produced by powdery mildew.

The stress response varied in the studied plants depending on the cultivation conditions. In the experimental plot, physiological reactions and the difference between transgenic and WT plants were more pronounced than in laboratory. Different cultivation conditions, including ultraviolet, X-ray and temperature stress, can change chromatin accessibility and define epigenetic regulation of gene expression, even when a constitutive promoter is used [32,58]. Moreover, it has been shown that plants contain more glucosinolates when grown in the field [59]. Consequently, field experiments are important when studying genetically-modified plants, as the results obtained in the laboratory cannot be extrapolated to agroecosystems.

Another interesting result of our study is the species-specific nature and diversity of powdery mildew species in the same field. Powdery mildews, identified as *E. convolvuli* and *E. cichoracearum*, were harmless to *B. napus*. This indicates that in the field, *B. napus* is primarily infected by overwintered mycelium and cleistothecia, and not conidia from infected weeds.

We have demonstrated that the exogenous expression of *GSTF11* can inhibit the growth of powdery mildew mycelium and reduce the mortality of transgenic plants. Based on research results and literature data, we suggest that increased content and activity of GST may reduce the death rate among infected plants by protecting distal healthy cells from damage. At the same time, ITCs in the apoplast may suppress the development of fungal mycelium [54,60,61]. However, only a fungistatic effect, and not a fungicidal one, was observed. Our results suggest that upon powdery mildew infection, GSTF11 contributes to an increase in GSH level and a decrease in GSH:GSSG ratio, which is indicative of excessive detoxification of the cell. These results could be additionally verified by measuring the content of the exact GSTF11 substrate. However, there is evidence that GSTF11 and other GST genes, upregulated by fungal infections and ITCs, are involved in the general stress response rather than a specific response. GSTs involved in GSL biosynthesis can be replaced by GSTs from other organisms [24,31,62]. Therefore, the creation of transgenic plants with exogenous expression of *GSTF11*, *GSTF6*, *GSTF10*, *GSTU20*, *GSTZ1* and other GST genes may help to define the role of GSL-related GSTs and lead to the production of stress-resistant plants for agriculture.

## 4. Materials and Methods

### 4.1. Biological Material

*B. napus* of the spring variety “Ratnik” was used in the experiments. The seeds were sown under natural conditions in the experimental plot and under laboratory conditions. The seeds were placed in vessels filled with commercial soil (Geolia, Russia). In the laboratory, plants were grown at 20 °C under 10,000 lux and a 16:8 h day:night photoperiod, generated by LED grow light. The experimental plot was located in the city of Ufa (54°43′34″N 55°56′51″E). Field experiments were carried out in August with an average daytime temperature of +17 °C, an average nighttime temperature of +16 °C, and an average humidity of 74%. According to the Hydrometeorological Center of Russia, the maximum temperature was + 26°C and the minimum was +10 °C.

Powdery mildew was isolated from naturally infected plants, co-occurring on the field (*B. napus*, *C. arvensis* and *S. oleraceus*).

### 4.2. Generation of Transgenic Plants

The *AtGSTF11* gene (At3g03190; NM_111189.3) was amplified from cDNA of *A. thaliana* using primers 5’-AGAAAATGGTGGTCAAAGTATATGG-3’, 5’-CGGAGGACTACAAGAACTACTAGACA-3’ and Pfu DNA polymerase (New England Biolabs, Ipswich, Massachusetts, USA). The resulting amplicon (678 bp) was cloned into a binary vector pCambia 1301 (CAMBIA, Australia) with a 35S promoter cassette [57,63] and *GUS* as the reporter gene.

Genetic constructs were cloned in *E. coli* XLblue strain and *A. tumefaciens* strain Agl0. *A. tumefaciens* carrying genetic construct 35S:AtGSTF11 were obtained by electroporation and used for in planta transformation of *B. napus* [64,65]. Agrobacterium suspension with a concentration of OD600 = 1, containing 30 g/L sucrose and 0.1% Silwet gold, was used for the inoculation of inflorescences. Treated plants were covered with plastic wrap for 24 h.

The seeds from the treated plants were soaked in 100 mg/L hygromycin for 24 h and planted in the soil. Seedlings showing chlorosis were removed, and the rest were subjected to PCR analysis and β-glucuronidase assay. GUS activity was evaluated by a histochemical method using X-Gluc reagent [66]. Basic morphological parameters were measured in WT and transgenic plants (stem length, leaf length and width, as well as the average weight of 100 seeds).

In each generation, transgenic plants were selected in the same way. Third generation of three stable lines of transgenic plants with increased *GSTF11* expression were used in experiments (lines 8, 30, 47).

### 4.3. Identification and Genotyping of the Pathogen

As an obligate biotrophic pathogen, powdery mildew cannot be stored in microorganism collection on culture media, and therefore, is not commercially available. To study the resistance of transgenic plants, it was necessary to isolate and distinguish among powdery mildew species. The pathogen was detected on the oilseed rape field at the end of August. The leaves, covered with white powdery growth, were taken from naturally infected *B. napus* and its weeds, *C. arvensis* and *S. oleraceus*.

Leaf samples were collected separately and used for DNA extraction. Fungi were washed from the leaves with distilled water. Then, 100 µL of the lavage was analyzed using *Biozero* BZ-8100 (Keyence, Osaka, Japan) microscope, and the rest was centrifuged in a 1.5 mL tube at 4000 *g*. The precipitate was disrupted with a micropestle and used for DNA extraction with DNeasy Plant Mini Kit (QIAGEN, Venlo, Netherlands). PCR for sequencing of ITS region was performed using primers 5′-GAGGCAATAACAGGTCTGTGATGC-3′ and 5′-CACCTCCTCCGCTTATTGATATGC-3′ [67]. PCR products were purified with diaGene Kit (dia-m, Moscow, Russia) and sequenced by Evrogen (Moscow, Russia) using Sanger method.

### 4.4. Nucleic Acid Extraction and Analysis

DNA was extracted from the first true leaves of the seedlings using the DNeasy Plant Mini Kit (QIAGEN, Netherlands). The presence of the genetic construct was confirmed using a primer for the 35S promoter (5′-GTGAAGATAGTGGAAAAGGAAGGT-3′) as Forward and a primer for *AtGSTF11* gene (5′-TAGAGCCACAGCGTAGAAATAGTT-3′) as Reverse. Positive plants were additionally screened for agrobacterial contamination, using primers for *rpoA* gene of *A. tumefaciens* (AF111855.1) 5′-TTCTGTTGTCTTCTCTGCGTGGTG-3′ and 5′-CGATTCTTCTTCTGCTTCCTTCTG-3′.

The expression of *GSTF11* gene was studied before and after each type of treatment. In the experiment with powdery mildew, samples were taken at the age of 30 days (before infection) and 60 days (after manifestation of powdery mildew growth). RNA was extracted from 50 mg of the leaf tissue using Lira reagent (Biolabmix, Novosibirsk, Russia). cDNA was synthesized using oligo(dT) primer from OT-1 kit (Syntol, Russia) and subjected to Real time PCR analysis using primers 5′-AGGTCAAGTTCGACAAGGTCC-3′ and 5′-CATACCGGGCATATGACTCAA-3′ for *AtGSTF11* gene. Due to the high level of homology between *AtGSTF11* and *BnGSTF11* (87% identity) it was impossible to design primers that would not give any product in nontransgenic plants. Therefore, mRNA content of both endogenous and exogenous *GSTF11* gene was evaluated.

*Actin7* (primers 5′-AGGAATCGCTGACCGTATGAG-3′ and 5′-GCTGAGGGATGCAAGGATGGA-3′) and *UBC9* (5′-GCATCTGCCTCGACATCTTGA-3′ and 5′-GACAGCAGCACCTTGGAAATG-3′) were used as reference genes [68]. Both genes were stably expressed in *B. napus* and *A. thaliana* at a high level. Reaction was set in M-427 master mix (Syntol, Moscow, Russia) containing SYBR Green dye using iQ5 detection system (Bio-Rad Laboratories, Hercules, California, United States).

### 4.5. Stress Treatment

To induce drought stress, 60 day-old plants cultivated in laboratory were deprived of water until wilting occurred (7 days).

Plants in the experimental plot were subjected to cold stress of natural origin in November (at the age of 200 days). RNA was extracted from plants when the outdoor temperature dropped to −1 °C, and then to −8 °C. Plants cultivated in the laboratory were not subjected to cold stress, because a sudden decrease of temperature rarely occurs in nature, and in our earlier studies, *GSTF11* expression level in *A. thaliana* did not change after a sudden drop of temperature from 22 °C to 8 °C. The 35S:AtGSTF11 transgenic hairy roots of tobacco at +3 °C were the same as in control plants [41].

To infect plants with powdery mildew, their leaves were rubbed with the freshly harvested leaf covered with white powdery growth to break up the conidial chains and transfer mycelium [22,69,70,71,72]. Control plants remained untreated.

Thirty plants of each transgenic line were infected in both the laboratory and plot experiments. Additionally, 100 WT plants were infected with powdery mildew in the experimental plot and 30 were infected in the laboratory.

The appearance of powdery mildew depends on the age of the plants, and can take up to 44 days [1]. After 30 days, treated and untreated plants from the laboratory and field experiments were used for RNA extraction and spectrophotometric assay of GST activity, GSH and GSSG content.

The disease assessment was carried out on five leaves per plant. Severity of infection was assessed by measuring the lesioned area using BZ-Analyzer software. After 100 days, the death rate of plants in each pot was evaluated.

### 4.6. Spectrophotometric Assay

Fresh leaves of *B. napus* were homogenized using Minilys homogenizer (Bertin Technologies, France) and simultaneously used to measure the activity of GST by spectrophotometric assay. GST activity was measured using the model substrate CDNB (138630, Sigma-Aldrich, Burlington, MA, United States) [37,73]. Protein concentration was determined by the Bradford method [74].

Levels of glutathione and glutathione disulfide were determined using o-phthalaldehyde (79760, Sigma-Aldrich, Burlington, MA, United States) as a fluorescent reagent, derivatization of GSH to prevent GSH autooxidation was performed using *N*-ethylmaleimide (E1271, Sigma-Aldrich, Burlington, MA, United States) [75]. GSH:GSSG ratio, indicating plant redox status, was measured as total glutathione (GSH+GSSG)/oxidized glutathione (GSSG).

All measurements were performed in six replicants in 96-well plates using a Perkin Elmer LS 55 Luminescence Spectrometer (Perkin Elmer, Waltham, MA, USA). The experiment was repeated in three technical and three biological replicates.

### 4.7. Statistical analysis

The size of the groups used in this study was variable. Most of the groups consisted of 30 plants (30 WT plants and 30 transgenic plants of each line). Due to the severity of stress at −8 °C, only 10 plants were in each group. The “susceptible” group of laboratory experiment included WT plants (*n* = 24) and transgenic plants in line 8 (*n* = 7), line 30 (*n* = 5) and line 47 (*n* = 9). In the same group as the plot experiment, there were 30 WT plants, 8 transgenic plants in line 8, 8 plants in line 30 and 5 plants in line 48. In the “resistant” group, there were 20 transgenic plants in each line (except for line 47 in the laboratory experiment, *n* = 18). There were 5 “resistant” WT plants in the laboratory and 15 in the plot experiment.

For all experiments, means and standard deviation (*p* < 0.05) were compared by analysis of variance (ANOVA) using LibreOffice v. 6.4.7.2. The calculation of Spearman’s correlation coefficients and the construction of correlation matrices were carried out using the DisplayR software. Correlation matrices are shown in Figure 7. Sequences were analyzed using MEGA 10.1.8 and aligned by ClustalW method. Phylogenetic trees were estimated from 1000 bootstrapped replications. Results of RT PCR were assessed by 2^−∆∆CT^ method.

## Data Availability

The data supporting the findings of this study are available within the article. The seeds of transgenic plants are stored in the collection of UFRC RAS and are available on request. Sequences of powdery mildew ITS marker are available at NCBI database (MW267299-MW267301).
